# Supplementary material for: From sequence to enzyme mechanism using multi-label machine learning
Source: BMC Bioinformatics. 2014 May 19;15:150. doi: 10.1186/1471-2105-15-150 (PMC4229970; doi:10.1186/1471-2105-15-150)
Supplement: Additional file 2 — Java code of ml2db. Additional file ml2db_code.tar.gz contains the Java source code to run the multi-label machine learning experiments and save the results to database. The code’s Javadoc is included. [file 1471-2105-15-150-S2.zip › additional file 2/ml2db/ml2db/doc/mulan/examples/SubsetLearnerExamples.html]

SubsetLearnerExamples


JavaScript is disabled on your browser.


- Overview
- Package
- Class
- Use
- Tree
- Deprecated
- Index
- Help

- Prev Class
- Next Class

- Frames
- No Frames

- All Classes

- Summary:
- Nested |
- Field |
- Constr |
- Method

- Detail:
- Field |
- Constr |
- Method


mulan.examples

## Class SubsetLearnerExamples

- java.lang.Object
- - mulan.examples.SubsetLearnerExamples

- ---

    

  ```
  public class SubsetLearnerExamples
  extends java.lang.Object
  ```

  A main class with examples for SubsetLearner, GreedyLabelClustering and
  EnsembleOfSubsetLearners methods usage.

  Version:
  :   2012.02.06

  Author:
  :   Lena Chekina (lenat@bgu.ac.il)

- - ### Constructor Summary

    Constructors

    Constructor and Description

    `SubsetLearnerExamples()`
  - ### Method Summary

    Methods

    | Modifier and Type | Method and Description |
    | --- | --- |
    | `static void` | `main(java.lang.String[] args)` Executes this example |

    - ### Methods inherited from class java.lang.Object

      `equals, getClass, hashCode, notify, notifyAll, toString, wait, wait, wait`

- - ### Constructor Detail


    - #### SubsetLearnerExamples

      ```
      public SubsetLearnerExamples()
      ```
  - ### Method Detail


    - #### main

      ```
      public static void main(java.lang.String[] args)
                       throws java.lang.Exception
      ```

      Executes this example

      Parameters:
      :   `args` - command-line arguments -path, -filestem,
          e.g. -path dataset/ -filestem emotions

      Throws:
      :   `java.lang.Exception`


- Overview
- Package
- Class
- Use
- Tree
- Deprecated
- Index
- Help

- Prev Class
- Next Class

- Frames
- No Frames

- All Classes

- Summary:
- Nested |
- Field |
- Constr |
- Method

- Detail:
- Field |
- Constr |
- Method
